# Incobotulinum Toxin-A Improves Post-Surgical and Post-Radiation Pain in Cancer Patients

**DOI:** 10.3390/toxins8010022

**Published:** 2016-01-13

**Authors:** Rezvan Rostami, Shivam Om Mittal, Reza Radmand, Bahman Jabbari

**Affiliations:** 1 Department of Neurology, Yale University School of Medicine, 15 York Street, LCI Building, New Haven, CT 06520, USA; rezvan.rostami@yahoo.com; 2 Department of Neurology, Case Western Reserve University School of Medicine, Cleveland, OH 44106-5040, USA; Shivam.Mittal@uhhospitals.org; 3 Department of Surgery, Bridgeport Hospital, 267 Grant Street, Bridgeport, CT 06610, USA; Reza.radmand@gmail.com

**Keywords:** incobotulinum toxin A, onabotulinum toxin A, radiation, Patients’ Global Impression of Change, surgery, cancer pain, visual analog scale

## Abstract

Cancer patients who undergo surgery or radiation can develop persistent focal pain at the site of radiation or surgery. Twelve patients who had surgery or radiation for local cancer and failed at least two analgesic medications for pain control were prospectively enrolled in a research protocol. Patients were injected up to 100 units of incobotulinum toxin A (IncoA) intramuscularly or subcutaneously depending on the type and location of pain (muscle cramp or neuropathic pain). Two patients passed away, one dropped out due to a skin reaction and another patient could not return for the follow up due to his poor general condition. All remaining 8 subjects (Age 31–70, 4 female) demonstrated significant improvement of Visual Analog Scale (VAS) (3 to 9 degrees, average 3.9 degrees) and reported significant satisfaction in Patients’ Global Impression of Change scale (PGIC) (7 out of 8 reported the pain as much improved). Three of the 8 patients reported significant improvement of quality of life.

## 1. Introduction

Surgery and radiation for cancer treatment has focal side effects on skin, soft tissues, muscles and nerves leading to muscle atrophy, fibrosis and contracture [1,2,3]. Approximately 30% of the patients with head and neck cancer and 20%–60% of the patients with breast cancer will have chronic pain localized to the site of radiation or surgery [4,5]. Treatment of post-surgical/post-radiation pain is difficult and failures are not uncommon. Moderate to severe postsurgical pain often requires opioids, which have abuse potential and in this context often fail pain management [6]. Local treatment for persistent pain with lidocaine patches and hyaluronic acid has provided only temporary relief [7,8]. 

Botulinum neurotoxins (BoNTs) block the release of neurotransmitters from presynaptic vesicles via inhibition of SNARE proteins, i.e. Soluble NSF (N-ethylmaleimide-sensitive factor) Attachment protein Receptor, which promote rupture of these vesicles [9]. The underlying mechanism of action of BoNTs in pain relief has not been yet elucidated convincingly. The animal data suggests blocking the release of number of pain modulators from presynaptic vesicles in addition to acetylcholine. Blinded clinical trials in humans indicate efficacy or probable efficacy in management of several pain disorders [10].

**Table 1:** Previous studies with OnaA and AboA for treatment of focal post-surgical radiation pain in cancer patients.

Author/Year	Total Patient (*n*)	Type of Study	Type of Cancer/Treatment	Type of Pain	Type of Toxin	Site of Injection	Total Toxin Dose (Unit)	Pain Scale	Outcome
Van Daele *et al*. 2002 [11]	6	Case series	Head and neck/radiation	Intermittent, tightness, Painful spasms	Toxin A (not specified)	Sternocleidomastoid muscle	40	No pain rating scale used	4 patients demonstrated complete pain relief
Wittekindt *et al*. 2006 [9]	23	Prospective	Head and neck/surgery	Spontaneous, continuous, burning, shooting, allodynia	AboA	Targeted areas of pain in the neck	80–240	Global quality of life scale, scale pain	Pain relief: 13 patients (56.5%) Pain unchanged: 6 patients (26%) Pain increased 4 patients (17.4%)
Hartl *et al*. 2008 [12]	19	Non-randomized prospective	Head and neck/radiation	Spontaneous, cramps, trismus	AboA and OnaA	masseter	250 (abo), 50 (ona)	Trismus-pain questionnaire	All showed significant improvement of overall functional score, muscle cramps and pain
Mittal *et al*. 2012 [13]	7	Retrospective	Lung, breast, head and neck	Neuropathic, myofascial	OnaA	Affected areas of pain (neck, breast, thorax)	20–80	VAS, PGIC scale, quality of life scale	Pain improved in all patients
Bach *et al*. 2012 [4]	9	Retrospective	Head and neck/radiation and surgery	Contracture	AboA	Sternocleidomastoid	100–800	Functional disability scales for neck pain	pain and neck motion improvement (7 patients), pain relief but no neck motion improvement (1 patient), No improvement (1 patient)
Dessy *et al*. 2014 [14]	1	Case report	Breast/surgery	Numbness, pins, burning	OnaA	Pectoralis major	50	Tinel’s sign and pain relief reported by patient	Complete pain relief, able to lift and rotate the arms, return to a normal personal and professional life

AboA = Abobotulinum toxin A, OnaA = Onabotulinum toxin A.

Pain relief after local injection of onabotulinum toxin A (OnaA) and abobotulinum toxin A (AboA) has been reported before in a small number of patients with post-radiation/post-surgical pain (Table 1) but not with incobotulinum toxin A (IncoA). In this communication, we report the results of a pilot, prospective study, which assessed the efficacy and safety of incobotulinum toxin A in focal cancer pain of 8 subjects after cancer surgery or radiation.

## 2. Methods

### 2.1. Participants

Adult subjects with cancer and focal chronic pain after surgery and/or radiation who have failed to respond to at least two analgesic medications were eligible to participate in the study. Participants were excluded if they had received prior botulinum toxin treatment, had diseases of neuromuscular junction or were on antibiotics or pharmaceutical agents with potential to affect neuromuscular transmission.

### 2.2. Study Design

The study was approved by the Yale Human Use Committee and registered at ClinicalTrials.gov (NCT01931865). Subjects were enrolled in the study after meeting the eligibility criteria and signing an informed consent. Demographic data, history, type of cancer, location of pain and type of medications were recorded along a neurological examination during the first visit (Table 2). The level of pain was measured by the visual analog scale (VAS 0–10) and improvement of the quality of life (QOL) was assessed by the American Chronic Pain Association questionnaire. The patients’ level of satisfaction with treatment was measured by Patients’ Global Impression of Change (PGIC) at the subsequent visits. Neurologist with over 20 years’ experience in botulinum toxin treatment (B.J.) performed the incobotulinum toxin A injections. When pain was in the form of muscle spasm, incobotulinum toxin A was injected intramuscularly. In case of neuropathic, superficial pain with typical burning quality the injections were subcutaneous and in a grid like pattern. The location and type of pain, pattern of injections and dose per patient is depicted in Table 2. The VAS and QOL were reassessed at baseline and at 4, 6, 8, and 12 weeks. Patients’ Global Impression of Change (PGIC) was also recorded at 4, 6, 8 and 12 weeks. The neurological examination was repeated at 6 weeks and 12 weeks. Patients’ medications during the study remained unchanged unless recommended by the treating oncologist.

### 2.3. Primary and Secondary Outcomes

The primary outcome in this study was improvement in pain as defined by 2 or more grade change in the VAS at 6 weeks after injection. The secondary outcomes were changes in the PGIC and QOL. The side effects were obtained and documented during each assessment.

**Table 2:** The patient’s demographic information, cancer type, prior treatment, and outcome before and after incobotulinum toxin A injection.

Pt No/Sex/Age	Location/Pathology of Cancer	Treatment and Medication before Botulinumtoxina Injection	Nature/Site of Pain	Sites/Total Dose of Injection (Unit)	Initial VAS/ VAS at 6 Weeks/ VAS at 12 Weeks	PGIC at 6 Weeks/PGIC at 12 Weeks
1/F/56	R-breast/adenocarcinoma	Surgery/Gabapentin, Lidoderm patch, Methocarbamol	Sharp, burning, superficial/R-upper abdomen, below rib cage	Subcutaneous, grid-like/100	10/5/7	Minimally improved/ minimally improved
2/M/60	L-tonsil/squamous cell carcinoma	Surgery/Morphine, Dilaudid	Sharp, superficial with allodynia, muscle spasms and tightness/L-temporal, L-zygomaticus and masseter	Subcutaneous, grid-like/95	10/5/7	Much improved/ much improved
3/M/31	R-frontal lobe/oligo-dendro-glioma	Craniotomy, radiation/Methadone, Depakote, Clonazepam	Sharp, burning, superficial/R-frontotemporal scalp, R-posterior neck, L-frontotemporal scalp, L-posterior neck	Bilateral subcutaneous/100	10/7/8	Much improved/ very much improved
4/F/70	R-breast metastasized to R-jaw/adenocarcinoma	Surgery/Gabapentin, Oxycodone, Ibuprofen	Dull constant/R-masseter, rizorius, zygomaticus	Subcutaneous divided into 5 sites/85	10/5/8	Much improved/ much improved
5/M/56	L-tonsil/squamous cell carcinoma	Surgery, radiation/None *	Severe, painful cramps/bilateral masseter	Subcutaneous both masseter, grid-like/100	10/3/8	Very much improved/ very much improved
6/F/51	R-breast/adenocarcinoma	Surgery, radiation/Gabapentin, Oxycodone	Dull, deep pain and muscle spasms/R-shoulder, arm, hand	Subcutaneous R-pectoralis, trapezius, triceps divided into 4 sites/100	5/3/3	Much improved/ very much improved
7/F/64	L-breast/inflammatory carcinoma	Surgery, radiation/Ibuprofen, Aspirin	Sharp, superficial/L-upper abdomen under L-breast	Subcutaneous, grid-like/100	9/0/0	Very much improved/ very much improved
8/M/53	R-neck/squamous cell carcinoma	Surgery, radiation/None *	Sharp muscle spasms/both masseters upper right, sternocleidomastoid	Subcutaneous divided into different units and sites/80	5/2/1	Very much improved/ very much improved

M: Male, F: Female; * These patient failed multiple medications before but at the time of enrollment were on no medications; VAS = visual analog scale, range from 0 (no pain) to 10 (severe pain); PGIC = Patients’ Global Impression of Change scale.

## 3. Results

A total of 25 patients were screened and 12 were enrolled in the study. Four patients did not finish the study. Two died because of advanced cancer, one could not return for follow up due to poor general health and one also did not return due to a skin reaction which developed shortly after incobotulinum toxin A injection. Of the remaining 8 who completed the study, four were female. The mean age was 55 years (range, 31–70 years). Patient’s demographic information is presented in Table 2. Incobotulinum toxin A was diluted with saline (1 cc per 100 units) and injected via a 27.5 gauge needle intramuscularly, subcutaneously (SC) or both depending on the nature of pain (SC for neuropathic pain with allodynia). The total dose per patient varied from 80–100 units. Table 2 provides information regarding the site of injections and dose of injected toxin per each muscle.

All patients demonstrated significant improvement in VAS (more than two grade improvement) at 6 weeks (Table 2). Five of 8 patients retained the same degree of improvement at 12 weeks. At 6 and 12 weeks, all patients reported their pain as improved with 7 of 8 reporting the pain as much improved or very much improved (Table 2). The quality of life (American Chronic Pain Association) improved in 3 of 8 patients (38%). The skin rash, which developed in that one patient, was maculo-papillary in nature and affected the upper torso on her back from T1 to T7 level bilaterally (not at the site of injection). It developed within few days after injection and gradually disappeared over 4 weeks. The cause and effect could not be clearly established. Other patients did not demonstrate any side effects after injections.

## 4. Discussion

Local post-surgical chronic pain occurs in 20%–70% of cancer patients [15]. The cause of chronic and maintained pain is believed to be due to peripheral receptor sensitization at the site of injury and central neuronal sensitization at spinal or supra-spinal levels. The peripheral noxious stimulus leads to local accumulation of the pain modulators (calcitonin gene related peptide, substance *P*, glutamate), which cause vasodilation and local inflammation. The inflammation leads to a series of complex reactions such as proliferation of CNS macrophages and impaired receptors inhibitory function. The nerve damage also increases sodium release and causes ectopic nerve discharge as a result of neuroma formation at the site of injury. These phenomena lead to sensitization of peripheral nerve endings and central sensitizations [16,17,18].

Radiation therapy has acute and delayed side effects the magnitude of which is dose dependent. Animal studies have shown focal loss of capillaries and muscle degeneration 2–4 months after a one-time 2000 rad (20 Gy) treatment. The damage increases collagen and decreases proteoglycans in extracellular matrix leading to disorganized structure and fibrotic tissue formation [11]. Focal pain is reported in about 20%–30% of patients after radiation therapy of head and neck cancer [12,19].

Data from animal studies have proposed a variety of mechanisms to explain the botulinum toxin (BoNT) induced analgesia after local injection. The data suggests that both peripheral and central mechanisms play a role in BoNT induced analgesia, although the exact mechanisms still not clear. Animal data demonstrates inhibition of substance P and calcitonin gene related peptide (CGRP) release from dorsal root ganglia (DRG) after peripheral injection [20,21] and modulation of sodium channels in peripheral and central nervous system [22]. An anti-inflammatory effect is strongly suggested for both OnaA and RimaB botulinum toxins in the formalin model of pain. Toxin injection into the paw of rat or mice 7–10 days before formalin injection significantly reduces the peak of inflammatory pain and local accumulation of glutamate in the injected area [23,24]. A central effect upon spinal neurons through retrograde transfer and transcytosis has been proposed by a number of investigators [25,26] including action via enhancement of Gabaergic mechanisms [27]. Blinded clinical trials have shown efficacy in some areas of human pain such as low back pain [28], plantar fasciitis [29], posttraumatic/post herpetic neuralgia [30] and migraine [31].

This is the first report on the efficacy and safety of incobotulinum toxin A in cancer patients with post-surgical and post-radiation pain. In this prospective study, all patients met the primary outcome *i.e.*, improvement of pain by two or more grades as assessed by VAS. Seven of the eight patients also reported significant satisfaction with treatment (secondary outcome-PGIC). The rather low number of patients (3 out of 8) who showed improvement of QOL in this study is in contrast with VAS and PGIC results. This is most likely due to the complexity of the quality of life questionnaire and to the fact that in patients with advanced cancer, many factors other than pain influence the quality of life. Considering the high incidence of placebo response in subjects with pain, the value of VAS in this population need to be evaluated in a blinded study. Incobotulinum toxin A treatment improved both muscle spasms and neuropathic pain (burning and jabbing pain). This finding is in agreement with the prior reports on the efficacy of other botulinum toxin as in management of this type of human pain (Table 1). Prior studies also reported no significant side effects with similar applied doses.

In conclusion, the result of our study and other open studies suggests the efficacy and safety of all three types of BoNT-A in alleviating the chronic pain in cancer patients after surgery or radiation. The limitation of these studies is the small number of the studied subjects and the open design of these studies. Blinded, placebo-controlled studies are necessary to support or refute the efficacy of botulinum toxin injections in relieving post-surgical or post-radiation pain in cancer patients.
